# Ovarian cancer screening—Current status, future directions^☆^

**DOI:** 10.1016/j.ygyno.2013.11.030

**Published:** 2014-02

**Authors:** Usha Menon, Michelle Griffin, Aleksandra Gentry-Maharaj

**Affiliations:** Gynaecological Cancer Research Centre, Women's Cancer, UCL EGA Institute for Women's Health, Maple House, 149 Tottenham Court Road, London W1T 7DN, UK

**Keywords:** Ovarian cancer, Screening, Biomarkers, Imaging, UKCTOCS

## Abstract

Evidence of a mortality benefit continues to elude ovarian cancer (OC) screening. Data from the US Prostate, Lung, Colorectal and Ovarian (PLCO) Cancer Screening Trial which used a screening strategy incorporating CA125 cut-off and transvaginal ultrasound has not shown mortality benefit. The United Kingdom Collaborative Trial of Ovarian Cancer Screening (UKCTOCS) is using the Risk of Ovarian Cancer (ROC) time series algorithm to interpret CA125, which has shown an encouraging sensitivity and specificity however the mortality data will only be available in 2015. The article explores the impact of growing insights into disease aetiology and evolution and biomarker discovery on future screening strategies. A better understanding of the target lesion, improved design of biomarker discovery studies, a focus on detecting low volume disease using cancer specific markers, novel biospecimens such as cervical cytology and targeted imaging and use of time series algorithms for interpreting markers profile suggests that a new era in screening is underway.

## Highlights

• Ovarian cancer screening using CA125 cut-off has shown no mortality benefit.
• Initial results using a time-series algorithm have been encouraging.
• Cancer-specific biomarkers, novel biospecimens, targeted imaging to detect precursor lesion/low-volume disease are likely to be central to future screening strategies.

## Introduction

There is growing evidence that screening can impact on cancer specific mortality. Many countries have national screening programmes for breast, bowel and cervical cancers with the latter associated with significant (50–90%) reduction in disease specific mortality [1]. Key to the success of these strategies has been an understanding of the natural history of the cancer and the existence of a precursor lesion. Evidence of a mortality benefit continues to elude ovarian cancer (OC) screening and the US Preventive Services Task Force (USPSTF) has recently reconfirmed their previous recommendation that it should not be undertaken in the general population [2]. However a number of novel insights in the last few years into disease aetiology, evolution and biomarker discovery suggest that a new era in screening is underway. The current article reviews the evidence from recently reported OC screening trials and explores the impact of the growing understanding of OC on future screening strategies.

## Current status

A number of large prospective trials have reported in the last few years (Table 1). The ovarian arm of the Prostate, Lung, Colorectal and Ovarian (PLCO) Cancer Screening Trial was a randomised controlled trial (RCT) of 68,616 women aged 55 to 74 of whom 30,630 underwent screening between 1993 and 2007. The women were screened using serum CA125 using a cut-off of ≥ 35 kU/l and transvaginal ultrasound (TVS) for 4 years followed by CA125 alone for a further 2 years. Positive results were evaluated and managed by the participants' physicians with no prescribed study protocol [3]. At a median follow-up of 12.4 years (25th–75th centile 10.9–13.0), 118 and 100 deaths were reported in the screening and control arm, respectively, with a mortality rate ratio of 1.18 (95%CI 0.91–1.54). Moreover, there was a high (15%) serious complication rate in women undergoing surgery for false positive findings [4]. Limitations of the trial include the use of a single threshold rule to interpret CA125 as women were returned to annual screening if CA125 was within normal limits (< 35 U/ml), lack of a central protocol driven management and the lengthy follow-up after screening was completed due to a significant healthy volunteer effect in trial participants [5]. The long follow-up resulted in 40.6% of women with OC in the study arm being diagnosed after the end of screening [6]. It was these findings that led to the USPSTF reconfirming that OC screening should not be undertaken in the low risk population. Despite these recommendations, a recent survey of US physicians indicated that one in three physicians believed that OC screening was effective and were likely to offer it to the women [7].

More encouraging data regarding survival was obtained from the Kentucky Screening Study, a single arm annual ultrasound screening study of 25,327 women [8]. At a mean follow-up of 5.8 years, the 5-year survival rates in women diagnosed with primary invasive epithelial OC (screen positives and interval cancers) in the screening study were significantly higher (74.8% +/− 6.6%) compared to women treated at the same institution during the same period who were not study participants (53.7% +/− 2.3%) [9]. However these rates are not comparable due to the ‘lead time effect’ of screening. In addition, as the Kentucky Study was not an RCT, it is likely that there was a significant ‘healthy volunteer effect’ that contributed to higher survival in those who participated [10]. This is supported by data from both PLCO Trial and the United Kingdom Collaborative Trial of Ovarian Cancer Screening (UKCTOCS), where in the control arm (no intervention), the all-cause mortality was less than half that expected due to a ‘healthy volunteer effect’ [5,11].

The Japanese Shizuoka Cohort Study of Ovarian Cancer Screening was an RCT of 82,487 low-risk postmenopausal women who were screened using an annual ultrasound and CA125 using a cut-off. The trial showed encouraging sensitivity (77.1%) and specificity (99.9%) with the screened women more likely to be detected at an early stage (63%) compared to the control arm (38%) [12]. The mortality effect has however not been reported as yet.

The largest screening trial to date is UKCTOCS, an RCT of 202,638 women from the general population randomised in 2001–2005 to no intervention (control) or annual screening using either transvaginal ultrasound (USS) or serum CA125 interpreted by a ‘Risk of Ovarian Cancer’ algorithm (ROCA) with transvaginal ultrasound as a second line test (multimodal screening, MMS). ROCA is a Bayesian algorithm [13] that compares the CA125 profile of cases to that of healthy women and incorporates age-specific incidence of OC in calculating risk. The closer the individual's profile is to the pattern of diseased women, the higher the estimated risk. During prevalence screening, both MMS and USS strategies had encouraging sensitivity for primary invasive epithelial ovarian/tubal cancers (89.5% and 75%, respectively). PPV was significantly higher in the MMS group (35.1% versus 2.8% in USS) resulting in lower rates of repeat testing and surgery [14]. This in part reflects the high prevalence of benign adnexal abnormalities and the more frequent detection of borderline tumours in the USS group. During incidence screening, although the specificity remained high, the multimodal strategy had superior sensitivity (88.6% vs 65.8%) and PPV (21.7% vs 5.8%) compared to the USS arm for detection of primary invasive epithelial ovarian and fallopian tube cancers [15,16]. The performance of the ultrasound strategy was similar between PLCO and UKCTOCS. The proportion of cases detected in Stage I/II was 47.1% at prevalence and 40.3% at incidence in the MMS arm and 50% at prevalence and 51.5% at incidence in the USS arm. Screening in the trial was completed in December 2011 and results of the mortality impact are awaited in 2014–5.

More recently, a single-arm US prospective study of 4051 low risk postmenopausal women aged > 50 screened using ROCA has reported detecting 4 women with invasive OC of 10 who underwent surgery during 11 years of screening. All screen-detected cancers were ‘early-stage high-grade’ invasive epithelial OC and no interval cancers were reported. This study provides independent validation of the UK findings of high specificity (99.9%) and PPV (40%) of ROCA [17].

In women at increased risk due to a family history or confirmed mutation in high penetrance genes such as BRCA1/2, annual screening with CA125 using a cut-off and TVS does not detect early stage cancers [18,19]. This was confirmed again by the recent report from Phase I of the UK Familial Ovarian Cancer Screening Study (UKFOCSS). Between 2002 and 2008, 3563 women underwent annual screening with serum CA125 and TVS. Whilst the sensitivity for detection of incident OC/FT cancer within a year of the last annual screen was high (81.3%–87.5% depending on whether occult cancers were classified as interval cancers or true positives), only 30.8% of screen-detected OC/FTCs were Stage I/II. There was a suggestion that strict adherence to annual screening had an impact as Stage ≥ IIIC disease was more likely (85.7% versus 26.1%; p = 0.009) in those who had not had screening in the year before diagnosis compared to those who had screening [20]. The preliminary findings led to UKFOCSS Phase II where annual CA125 screening was replaced by 4-monthly serum CA125 interpreted using the ROCA. The preliminary results presented at the American Society of Clinical Oncology meeting in 2013 suggest that this strategy had high (67–100%) sensitivity for ovarian and tubal cancers, with no interval cancers reported. In Phase II, 42% of incident screen-detected OC/FT cancers were Stage I/II. However, 92% of incident screen-detected cancers were completely cytoreduced compared to 62% on Phase I (p = 0.16). Whilst the results were encouraging, it is important to note that screening at present cannot be considered a safe alternative to risk-reducing surgery [21]. A similar strategy was also assessed prospectively in the US screening trials in high-risk women undertaken under the auspices of the Cancer Genetics Network [22] and Gynaecology Oncology Group [23]. Screening is complete in all of these trials with results expected later this year.

## Lessons from past biomarker failures

In the past decade, major efforts have been made to improve on the performance of CA125 in differential diagnosis of pelvic masses and screening. Despite a plethora of biomarkers being investigated, CA125 remains the single-best biomarker for OC. In a study of 35 of the most promising OC biomarkers, using samples from the PLCO Trial bank taken from 118 women within 6 months prior to OC diagnosis and 951 age-matched controls, CA125 had the best sensitivity at 86%, with Human Epididymis Protein 4 (HE4) the second best performing marker with a sensitivity of 73% [24]. HE4 has so far not been approved for use in screening, although a commercial test, Risk of Ovarian Malignancy Algorithm, ROMA, that combines HE4, CA125II and menopausal status is in use for pre-operative triaging of women [25]. The performance of all markers was poorer in the PLCO study when assayed in samples taken more than 6 months from diagnosis [26]. Further studies found no improvement in performance over CA125 alone of a comprehensive panel that included 28 of the most promising OC biomarkers or smaller panels of 6 to 8 biomarkers [26] in samples within 12 months of diagnosis.

This failure to find useful biomarkers, despite major investment and research has led to explorations of the possible causes [27,28]. A lack of rigour in all three phases of biomarker discovery and validation has been identified; preanalytical—case selection, matching of controls and sample processing and storage, analytical—detection limit and precision of assays, and post-analytical—overfitting and data interpretation to yield ‘the next promising’ marker. There is a growing consensus that biomarkers discovered in clinical sample sets collected at diagnosis from symptomatic patients and controls in hospital settings may not be representative of the screening population. As a result, a key recommendation that has emerged is the use of the PRoBE (prospective-specimen collection, with retrospective-blinded evaluation) design for biomarker discovery and validation [29]. This involves blinded case control studies nested within a prospective cohort that represents the target population where biologic specimens and clinical data have been collected prior to ascertainment of outcome such as OC diagnosis on follow-up.

Increasingly samples predating diagnosis (preclinical/prediagnostic) are available from the biobanks built during the course of large cohort studies and screening trials. In a nested case control study using preclinical samples from the Carotene and Retinol Efficacy Trial (CARET) biobank, a panel of CA125, HE4, and mesothelin was found to provide a signal three years before OC diagnosis [30] whilst a nested case control study from UKCTOCS reported elevation of Putative Platelet Factor 4 (PF4) and connective tissue-activating peptide III (CTAPIII) 11 and 15 months respectively before OC diagnosis and before the rise in CA125 [31].

## Defining the target lesion—new insights into carcinogenesis

Malignant ovarian neoplasm, coded C56 by the International Classification of Diseases and Related Health Problems 10th revision code (ICD-10), is a heterogeneous group of ovarian tumours that include primary invasive epithelial, borderline epithelial (LMP) and non-epithelial OC. Over the years, significant confusion has existed as to which of these subtypes of OC constitute a valid target for ovarian cancer screening. More recently there seems to be growing consensus that it should be restricted to primary invasive epithelial OC as it is the main cause of disease mortality. Increasing evidence that primary invasive epithelial ovarian, fallopian tube (ICD-10 C57.0) and primary peritoneal (ICD-10 C48) carcinomas are Müllerian in nature has led to debate as to whether they represent a single disease entity ‘pelvic serous cancer’ [32,33]. This would suggest the widening of the spectrum of targeted cancers in OC screening. In UKCTOCS, the strategy focused on detecting primary tubal cancers in addition to malignant ovarian neoplasms [14], whilst in the PLCO Trial primary peritoneal cancers were also included in the primary outcome measure [4].

Based on distinctive morphologic and molecular genetic features, ‘invasive epithelial’ OCs can broadly be classified into two groups [34]. Type I are slow growing cancers with better prognosis such as low-grade serous, low-grade endometrioid, clear cell, mucinous and transitional (Brenner) carcinomas. These tumours generally behave in an indolent fashion, are confined to the ovary at presentation and are relatively genetically stable. They lack mutations of TP53 with each histologic type exhibiting a distinctive molecular genetic profile. They share lineage with the corresponding benign cystic neoplasm, often through an intermediate (borderline tumour) step, supporting the morphologic continuum of tumour progression. Endometrioid and clear cell tumours are associated with endometriosis [35] and many consider the endometrium to be the source of these ovarian neoplasms [34]. Preliminary data suggest that mucinous and transitional (Brenner) tumours arise from transitional-type epithelial nests at the tubal–mesothelial junction by a process of metaplasia [34]. Ultrasound screening appears to have higher sensitivity in picking up these cancers in comparison to Type II cancers (unpublished data from UKCTOCS).

Type II cancers include high grade serous, high grade endometrioid, undifferentiated tumours and carcinosarcomas. They are more aggressive and present mostly in late stage [34]. There is mounting descriptive molecular pathology and experimental evidence that a significant proportion of these cancers start as premalignant serous tubal intraepithelial cancer (STIC) lesions in the fimbrial end of the fallopian tube. STICs are composed of “secretory cells”, the non-ciliated population of the endosalpinx and most show strong nuclear staining with antibodies to p53 as well as an increased proliferation index (MIB-1) compared to the background tubal epithelium [36,37]. Several studies starting with the report by Kindelberger et al. [38] have shown that 33–59% of high-grade serous ovarian and primary peritoneal cancers co-exist with STIC lesions. In addition, precursor tubal lesions termed “p53 signature” have been found in one third of all women and are believed to represent the initial events of serous carcinogenesis—DNA damage of secretory cells and p53 mutations. These insights open up the prospect of ovarian cancer screening becoming more akin to cervical cancer screening with the potential to impact on cancer incidence by intercepting precursors of pelvic serous cancer. Much depends on discovery of novel detection methods using exfoliative cytology or imaging to detect these early lesions. Unanswered questions include the time interval before early serous cancers metastasise to peritoneal surfaces and whether they are more curable than advanced malignancies [37].

## Inferences from mathematical modelling

On the former front, after decades, progress is being made. Modelling using data from published series of occult serous OCs detected at risk reducing surgery in BRCA1 carriers has yielded some critical insights [39]. It is likely that serous cancers spend, on average, greater than four years in situ, Stage I or II and possibly a further one year as Stage III or IV before they present clinically. During this occult period the cancer is less than 1 cm in size, increasing to just 3 cm when it progresses to an advanced Stage III or IV [40]. There is then explosive growth with tumours doubling in volume every two and a half months. This suggests that to achieve 50% sensitivity in detecting tumours before they advance to Stage III, an annual screen would need to detect tumours of 1.3 cm in diameter.

These findings suggest that an annual screen could be a viable screening strategy given the relatively long ‘window of opportunity’ for detection prior to progression to Stage III. However a test that is sensitive and specific enough would need to detect tumours hundreds of times smaller than clinically apparent serous cancers. Overcoming the inherent signal-to-noise problem will require development of novel approaches beyond traditional blood biomarkers—discovery of truly cancer-specific molecules and use of alternative bio-specimens such as endocervical swabs, uterine or tubal lavage which would boost signal to noise by both reducing background from nonmalignant tissues and avoiding the problem of biomarker dilution inherent in blood-based assays. The data also lends further support to the fact that current screening strategies are more likely to detect low volume high grade serous (Type II) cancers rather than early stage.

For blood-based biomarkers, Hori and Gambhir undertook mathematical modelling to describe dynamic plasma biomarker kinetics in relation to the growth of a tumour, beginning with a single cancer cell. They used ovarian carcinoma progression and CA125 shedding in the average female patient to build the model. The results suggest that current blood assays can only detect tumours 9 to 10 years following the appearance of the first cancer cell at spherical volumes of 25 mm^3^ or more [40]. To detect tumour at sizes in the millimetre diameter range before metastasis, requires extremely high rates of biomarker secretion by tumour-associated cells, highly accurate assays with much lower detection limits than currently in clinical use and essentially zero background shedding from healthy cells so that baseline levels in women without OC are consistently very low.

## Cancer specific biomarkers

Cancer-related genes such as TP53, BRAF and KRAS are frequently mutated in OC with TP53 mutated in almost all Type II cancers whilst mutations in BRAF and KRAS are more common in borderline ovarian tumours and Type I cancers. It has been shown that small amounts of mutant alleles in cell-free body fluids can be quantified with unprecedented sensitivity by new technologies such as BEAMing [41]. Recently Forshew and coauthors reported identifying mutations in *TP53* at allelic frequencies of 2% to 65% in plasma from patients with advanced OC who had high levels of circulating tumour DNA (ctDNA) using tagged-amplicon deep sequencing (TAm-Seq). Through several experiments, the authors were able to show that TAm-Seq is a viable method for sequencing large regions of ctDNA. Although this provides a new way to noninvasively identify gene mutations in blood, TAm-Seq will need to achieve a more sensitive detection limit (< 2% allele frequency) to identify mutations in the plasma of patients with less advanced cancers. Nevertheless, once optimised, this low-cost, high-throughput “liquid biopsy” approach may allow detection of small tumours [42]. In the future, it would be crucial to assess the TP53 ctDNA signature in healthy controls.

An equally promising and novel approach was reported by Kinde et al. who developed a sensitive massively parallel sequencing method to test for mutations in a panel of 12 genes. When applied to 14 liquid cytology cervical samples from women with OC who had mutations, they were able to identify the expected tumour-specific mutations. However, the limitation of the study is that all specimens were from women with advanced stage disease; the utility of this approach in early stage disease is yet to be determined. The results demonstrate that in a proportion of OCs, tumour DNA can be detected in a standard liquid-based cervical cytology specimen obtained during routine pelvic examination [43]. Further improvements in the technology for e.g. increasing the number of potential gene targets could increase the technical sensitivity of the test whilst improved collection methods such as a small cannula introduced into the endometrial cavity, similar to the Pipelle endometrial biopsy instrument, could theoretically allow a more highly enriched sample of cells coming from the fallopian tube and ovary.

Autoantibodies warrant further evaluation as OC biomarkers as they could amplify the signal and improve lead time over CA125.

## Longitudinal algorithms

Equally important as the biomarkers themselves, is how results are interpreted. There is good evidence in the case of biomarkers that are not cancer specific such as CA125, single threshold rules used for diagnostic tests are not effective in the context of screening whether in the high or low risk population. Serial samples are an integral part of screening and algorithms incorporating change in an individual's marker profile over time as the cancer evolves have superior sensitivity and specificity. Retrospective analysis of PLCO Trial data showed that the CA125 velocity was a statistically significant predictor of OC with average velocity in those with cancer (19.749 U/ml per month) being more than 500 times that (0.035 U/ml per month) in women who did not have cancer [44].

The first of such time series algorithms was ROCA detailed previously, which was developed in the early 1990s [45]. Following a successful initial pilot [46], it is now being assessed in screening trials both in the high [21,23] and low risk populations [14,17]. It has been shown to significantly improve screening performance compared to a fixed cut-off for CA-125 [47]. Preliminary data from UKCTOCS presented at the Helene Harris Ovarian Cancer meeting in 2010 [48] showed that serial CA125 monitoring using the ROC algorithm can detect OC at tumour sizes too small to allow detection by transvaginal ultrasound. Between 17th April 2001 and 30th June 2008, in the multimodal arm of the trial, 147 women with no previous history of cancer were classified by the ROC algorithm to have risk of OC ≥ 1 in 5 despite two normal or unsatisfactory transvaginal scans. 15 of the 147 (11.4%) women were found to have ovarian/tubal/peritoneal malignancies. In the early years of the trial, there were delays in undertaking surgery due to reluctance on part of the clinicians to operate in the absence of imaging abnormalities or symptoms. This suggests that alongside refining such algorithms there needs to be a change in the clinical paradigm of what an early invasive epithelial OC looks like.

Other longitudinal algorithms such as the parametric empirical Bayes (PEB) longitudinal screening algorithm have also been shown to pick up OC earlier than the single threshold rule in PLCO samples [49]. As panels of biomarkers complementary to CA125 are assembled, further development of such algorithms to take into account the combined profile will be required to ensure detection of OC at low volumes. The serial samples available in screening trial biobanks are crucial in developing and validating such algorithms, which may improve the performance of existing markers.

## Real time imaging for cancer screening

Tumour angiogenesis is one of the hallmarks of cancer and is present early during the development and growth of different solid tumours. However initial studies using colour flow Doppler to analyse blood flow to suspicious areas or masses were not found to significantly add to assessment of malignant lesions [50]. Recently, insights into the intricacies of neovascularisation have highlighted a possible oversight that the microvascular rather than macrovascular pattern may be more significant. Using contrast enhanced transvaginal ultrasound with microbubbles that are small enough to pass through capillaries, the kinetics of the blood flow can be measured and analysed to detect abnormal flow found in areas of neovascularisation [51]. Microbubble-enhanced transvaginal sonography is being used to enhance the evaluation of ovarian masses by early detection of tumour microvascularity. However in due course it may be part of the screening protocol to identify OCs in women found to be at increased risk through rising serum marker profiles.

A more powerful imaging tool is targeted contrast-enhanced ultrasound imaging which has the potential for the detection and quantification of tumour angiogenesis at the molecular level. In a recent study, ultrasonographic microbubbles targeted with one of several antibodies, anti-integrin, anti-endoglin, or anti-vascular endothelial growth factor receptor 2 were injected into mice with implanted breast, ovarian, or pancreatic tumours. As the tumours grew, changes in the relative uptake of each targeted microbubble were observed, opening up the possibility of noninvasive in vivo molecular profiling of tumour angiogenesis as a diagnostic tool [52].

Another area being explored is light-induced intrinsic tissue fluorescence or autofluorescence (AF) that may be lost in cancerous/precancerous epithelial tissue. Assessment of AF enables real time, high resolution imaging of epithelial tissue and coupled to handheld/endoscopic devices, it has proven successful in early detection of cervical, skin, oral and oesophageal cancer. McAlpine et al. have reported using ex vivo reflectance and autofluorescence optical imaging of tubes removed at surgery to identify STIC lesions with promising levels of sensitivity (73–100%), specificity (83–92%), PPV (50–78%) and NPV (91–100%). Future developments include improving predictive ability and introducing in vivo real time imaging of the fallopian tube lumen using endoscopy [53].

## Defining the target population

Risk stratification improves the effectiveness of screening by enriching the population for individuals at greatest disease risk. Currently age and family history of ovarian, breast and colon cancers are used to define the low- and high-risk populations, eligible for OC screening. This is likely to evolve given the significant progress made in the last decade in understanding risk conferred by genetic and epidemiological factors. There is now good evidence that mutations in the high-penetrance susceptibility genes (BRCA1, BRCA2 and DNA mismatch repair genes) only represent an extreme end of a wide spectrum of OC genetic risk and that several other susceptibility genes exist. The impact of mutations in these genes is dependent on their minor allele frequencies and the magnitude of the allelic effect. Mutations in several moderate risk genes for e.g. RAD51C, RAD51D, and BRIP1 and multiple low risk (low penetrance) genes could account for the remaining excess familial risk. In addition, the genome-wide association studies have so far uncovered eight susceptibility loci for serous epithelial OC [54–58]. These loci harbour low-penetrance alleles with allelic odds ratios of less than 1.5. As these loci still explain only a small part of the heritable fraction, further large-scale studies are underway and it is likely that these numbers will increase [59]. Recently, Pharoah et al. described the potential value of using multiple, low-penetrance susceptibility alleles to refine risk stratification in the context of breast cancer screening strategies [60]. It is conceivable that a similar ‘polygenic’ approach could be applied to OC. Combining risk related to the genetic variants in these recent genome-wide association studies and several well-established environmental risk factors suggests that a multiplicative model is the best fit [61]. Although the future approaches to screening may include risk prediction modelling that considers both environmental and genetic risk factors (both germ line and somatic changes), the models would be complex and likely need validation in prospective trials. The implementation of such risk-stratified screening will eventually depend on a wide array of organisational, ethical, legal and social factors in addition to usefulness and cost-effectiveness [62].

## Conclusion

During the last decade, better understanding of the heterogeneity of OC and insights into its evolution have clarified the target lesion—‘primary invasive epithelial ovarian, tubal and peritoneal cancer and premaligant lesions such as STIC’. Past failures have led to growing numbers of biomarker discovery studies using a blinded case control design nested within population cohorts and focusing on individual OC subtypes. Modelling has provided insights into the natural history of serous OC and highlighted the need for highly accurate assays of cancer specific biomarkers using novel biospecimens such as cervical cytology that can detect low volume tumours. This has translated into early ‘proof of principle’ tests involving deep sequencing technology for mutations in specific genes associated with in-situ or low volume disease in tumour DNA isolated from such specimens or more traditional blood samples. Another key factor that has emerged is the importance of longitudinal algorithms in improving biomarker performance in the context of screening.

There was no mortality benefit of ovarian cancer screening in the PLCO trial. However the longitudinal algorithm ROCA used in UKCTOCS has shown encouraging performance characteristics both on prevalence and incidence screening. Preliminary data from the trial suggests that CA125 rise within normal range can be detected by the ROCA well before any abnormalities are detected on transvaginal imaging. Whether this converts into a mortality impact will only be known in 2015. Meanwhile major efforts are underway to improve OC risk stratification and identify populations at greatest risk of disease.

## Conflict of interest

UM has a financial interest through UCL Business and Abcodia Ltd. in the third party exploitation of clinical trial biobanks which have been developed through the research at UCL. None of the other authors have any conflict of interest; no other relationships or activities that could appear to have influenced the submitted work.

## Figures and Tables

**Table 1:** Summary of the key findings of the four major ovarian cancer screening trials.

	Ovarian cancer screening trials in the general population			
	University of Kentucky Study [8,9]	Japanese Shizuoka Cohort Study of Ovarian Cancer Screening [12]	Prostate, Lung, Colorectal and Ovarian (PLCO) Cancer Screening Trial [3,4]	United Kingdom Collaborative Trial of Ovarian Cancer Screening (UKCTOCS) [13,15,16]
Study design	Single arm prospective study	RCT with 1 screening strategy in study group	RCT with 1 screening strategy in study group	RCT with 2 screening strategies in the study group
Cohort	25,327	41,688	30,630	98,305
Screening strategy	Ultrasound	Physical exam, ultrasound and CA125	Ultrasound and CA125	Two screening arms (ultrasound, USS) and CA125 followed by ultrasound (multimodal, MMS)
Interpretation of CA125		CA125 using a 35 kU/l cut-off	CA125 using a 35 kU/l cut-off	CA125 interpreted using the Risk of Ovarian Cancer (ROC) algorithm
Key screening findings	Encouraging sensitivity (81%) for primary OC/FT cancer; 76.3% for primary invasive OC/FT cancer	Encouraging sensitivity (77.1%) for primary OC/FT cancer	Lower sensitivity (69.5%) for primary OC/FT cancer; 68.2% for primary invasive OC/FT cancer when compared to the other trials (only 28% were Stage I/II)	Encouraging sensitivity (89.4% MMS/84.9% USS) for primary OC/FT cancer; 84.9% MMS/75.0% USS for primary invasive OC/FT cancer (47% MMS/50% USS were Stage I/II). Superior sensitivity (88.6% vs 65.8%) and PPV (21.7% vs 5.8%) of MMS compared to the USS arm for detection of primary invasive epithelial OC/FT cancers during incidence screening, with 40.3% in the MMS and 51.5% in the USS arm detected at early stage.
Key mortality/surrogates of mortality findings	Longer 5-year survival in the screened arm (74.8%) compared to unscreened women from the same institution treated by the same surgical and chemotherapeutic protocols (53.7%) (p < 0.001).	Stage shift: more Stage I ovarian cancers in the screened group (63%) compared to the control (38%)	No mortality benefit: 118 ovarian cancer deaths in the screened arm, 100 in the control arm	Mortality data awaited in 2014/2015
Current status	Completed	Mortality data to be reported	Completed	Mortality data to be reported in 2015
